# Regional variation in urinary catheter use in the Netherlands from 2012 to 2021: a population-based cohort

**DOI:** 10.1177/17562872231215181

**Published:** 2023-11-30

**Authors:** Felice E. E. van Veen, Jeroen R. Scheepe, Bertil F. M. Blok

**Affiliations:** Department of Urology, Erasmus Medical Center, Room Na-1524, Dr. Molewaterplein 40, Rotterdam 3013 GD, The Netherlands; Department of Urology, Erasmus Medical Center, Rotterdam, The Netherlands; Department of Urology, Erasmus Medical Center, Rotterdam, The Netherlands

**Keywords:** clean intermittent catheterization, indwelling catheterization, practice variation, prevalence, underactive bladder, urinary catheterization, urinary retention

## Abstract

**Objectives:**

Our aim was to evaluate trends and regional differences in the use of indwelling and intermittent urinary catheters in the community setting in the Netherlands from 2012 to 2021.

**Design and methods:**

For this population-based cohort study, data on catheter use was collected from the Drug and Medical Devices Information System of the National Healthcare Institute of the Netherlands. This database contains information on the Dutch insured population, which was 100% of the total population in 2018. Users were divided into 12 provinces according to the Nomenclature of Territorial Units for Statistics codes. The number of catheter users was adjusted for the total population of the provinces by sex and age, and was expressed by users per 100,000 people. Negative binomial regression (NBR) was used to test for differences in clean intermittent catheter (CIC) and indwelling catheter (IDC) users across Dutch provinces.

**Results:**

Between 2012 and 2021, IDC users increased by 44.6% from 41,619 to 60,172, and CIC users increased by 27.3% from 34,204 to 43,528. The greatest increases were mainly observed among IDC users over 85 years old and male CIC users over 65 years old. NBR showed significant differences for IDC and CIC users between the 12 provinces. CIC incidence was higher in Drenthe and Groningen (Northern Netherlands) compared to Zuid-Holland (Southern Netherlands). IDC incidence was higher in seven provinces dispersed throughout the Netherlands compared to Noord-Holland.

**Conclusion:**

CIC and IDC users have continued to increase in recent years; this was especially observed among older men. In addition, there were regional differences in the number of CIC and IDC users; CIC was more prominent in the northern region of the Netherlands, and IDC varied between multiple provinces. Practice variation in urinary catheterization may result from patient population differences or healthcare provider preferences and their alignment with guidelines.

## Introduction

Indwelling urinary catheters are commonly used for urinary bladder drainage in the management of urinary retention. Urinary retention is the inability to (completely) empty the urinary bladder with or without the presence of bladder outlet obstruction. Urinary retention can be neurogenic or non-neurogenic in origin. The most common associated neurogenic causes are spinal cord injury, spina bifida, multiple sclerosis, and Parkinson’s disease.^1^ Non-neurogenic causes include bladder outlet obstruction (e.g. benign prostatic hyperplasia, urethra stricture), post-partum, post-pelvic surgery, or may be idiopathic in nature.^2^ It is important to differentiate between neurogenic and non-neurogenic causes of urinary retention since their treatment approaches differ fundamentally and may affect the choice of catheterization.

In addition, indwelling catheters (IDCs) can be used in the management of urinary incontinence. Urinary incontinence is a common condition that affects millions of people worldwide and can significantly impact their quality of life.^3^ There are several treatment options for this condition, including pads, urinary catheters, medication, or surgery.^4,5^ IDCs are particularly used in vulnerable patients with complex medical conditions or who cannot perform or tolerate other treatment options. Immobile and fragile patients often receive IDCs because of the increased risk of pressure ulcers and the high workload for caregivers associated with the use of pads. IDC provides continuous urinary drainage, which improves comfort and reduces the risk of pressure ulcers. However, the use of IDCs is not without risks. It can lead to complications such as catheter blockage, discomfort (bladder spasms), urethral injury, catheter-associated urinary tract infections and bladder stones.^6^

Clean intermittent catheterization (CIC) is considered the method of choice for bladder drainage in neurogenic and non-neurogenic patients.^7,8^ CIC involves the insertion and removal of a plastic disposable catheter to empty the bladder with an average of 4–6 times a day. Compared to IDCs, CIC reduces the risk of catheter-associated urinary tract infections, discomfort (bladder spasms), bladder stones, and renal deterioration, while increasing quality of life through greater independence, mobility, and maintaining the ability to engage in sexual activity.^9,10^ The decision to use either indwelling or intermittent catheterization depends on several factors, including patient factors (e.g. underlying disease, patient preference, hand function, and position of urethral meatus), and the availability of healthcare resources and reimbursement for disposable urinary catheters. Ultimately, the decision should be made on an individual basis, taking into account the patient’s individual needs and circumstances. However, the current decision-making process regarding assisted bladder drainage is not transparent or standardized. The choice of catheter type or the recommendation of alternative treatment options depends on the preference of medical professionals (physicians and specialized nurses), which is usually based on clinical experience combined with the acquaintance of specific manufacturers.

In the Netherlands, the use of CIC and IDC has increased substantially in the past two decades.^11,12^ However, it remains unknown whether there are regional differences regarding IDC and CIC users due to differences in prescribing behavior of medical professionals regarding urinary catheters. Only one previous study in England reported on the prevalence of long-term IDC use in the community setting in different regions of the country. They found a similar prevalence of IDC use in both the south (0.146%) and west (0.141%) of England.^13^ This study investigated the trends and regional differences in IDC and CIC users in the community setting (non-hospitalized and non-institutionalized) in the Netherlands over the past 10 years (2012–2021) with the use of combined national databases. Our hypothesis was that the number of IDC and CIC users has continued to increase equivalently in recent years. In addition, we hypothesized that there were no regional differences in the number of IDC and CIC users in the Netherlands, because the Netherlands is a small country with a uniform urology residency program, and because of adherence to professional guidelines by urologists and rehabilitation physicians for the preferred use of CIC.

## Methods

### Study design

For this retrospective, population-based database study, data were collected from the Drug and Medical Devices Information System (Genees- en hulpmiddelen Informatie Project; GIP) of the National Healthcare Institute in the Netherlands (Zorginstituut Nederland). The GIP database contains information on all reimbursed prescriptions from general practitioners and physicians for medication and medical devices in the community setting (non-hospitalized and non-institutionalized) in the Netherlands. Since 2006, the Health Insurance Act was introduced in the Netherlands, making data on the total insured population available, which has increased from 16.2 million (99% of the Dutch population) in 2006 to 17.1 million people (100% of the Dutch population) in 2018. Data are based on the number of prescriptions per patient per year. All data used were obtained and handled according to Dutch privacy laws.

The following data were evaluated for urinary catheter use by year:

(1) Number of IDC and CIC users of the total Dutch population from 2012 to 2021;
(2) Sex and age distribution of IDC and CIC users of the total Dutch population in 2012 and 2021;
(3) Number of IDC and CIC users per 12 Dutch provinces from 2012 to 2021;
(4) Sex and age distribution of IDC and CIC users by 12 Dutch provinces in 2021.

In the Netherlands, all declarations of medical devices by pharmacists or medical device suppliers are coded through ZI-numbers or Generic Product codes for devices (Generieke Product codes Hulpmiddelen; GPH). The ZI-numbers are assigned by Z-index in the G-Standaard database, which contains product information of medicines and medical devices that are dispensed by the Dutch health care system.^14^ The GPH-codes are managed by Vektis, a non-commercial database that is responsible for transmitting pseudonymized data from healthcare insurers to the National Healthcare Institute.^15^ The health insurers provide this information on declarations to the GIP database. The GIP database links the ZI-numbers and GPH-codes to a corresponding ISO9999-code that is translated into a classification. All urinary catheters are classified under the monitor code ‘A1535 catheters’ and are subcategorized by different ISO-codes. For this study, data were obtained from the ISO-codes for IDCs and disposable intermittent catheters (e.g. disposable intermittent catheters are ISO92406).

### Data analysis

For this study, all links between ZI-numbers/GPH-codes and ISO-codes were analyzed and checked by visual control of the product names. In addition, all occurring ZI-numbers were checked with the product information in BeverOnline, a medical device database from Nigella IT.^16^ Incorrect links between ZI-numbers/GPH-codes and ISO-codes or incorrectly classified products were removed. An improved classification was provided for medical devices that were previously misclassified. All individual catheter users were assigned a unique pseudonymized number linked to specific ISO-codes. After reclassifying the different medical devices, we categorized them into IDC and CIC users. IDC and CIC users were classified by sex (male and female), age categories (0–45, 45–65, 65–85, >85 years), and 31 regions of care administration offices. There are 31 regional care administration offices in the Netherlands that work on behalf of the health insurers within a region consisting of multiple municipalities. To analyze regional differences in catheter use, the 31 care administration office regions were subdivided into 12 provinces according to the Nomenclature of Territorial Units for Statistics (NUTS) codes of the Netherlands.^17^ If a care administration office consisted of municipalities from multiple provinces, the care administration office was assigned to the province to which most municipalities belonged. For an equivalent comparison of catheter users between provinces, the number of catheter users was adjusted for the average population of the provinces by sex and age, using population data from Statistics Netherlands (Centraal Bureau voor de Statistiek; CBS).^18^ The number of urinary catheter users was expressed by users per 100,000 insured people in the same specific age and sex category. Negative binomial regression (NBR) models were used to test for differences in IDC and CIC users across provinces. NBR was chosen because the assumptions of the Poisson regression model were not met due to overdispersion in the count data. Multivariable models were fit to account for the following characteristics: sex, age, and provinces. The age group 0–45 years was not included in the NBR analysis of IDC users, because the small number of count data in this group would lead to an unreliable model. All analyses were performed using SPSS Statistics version 28.0 (IBM Corp., Armonk, NY, USA).

## Results

### Catheter users: total population

**Figure 1.**
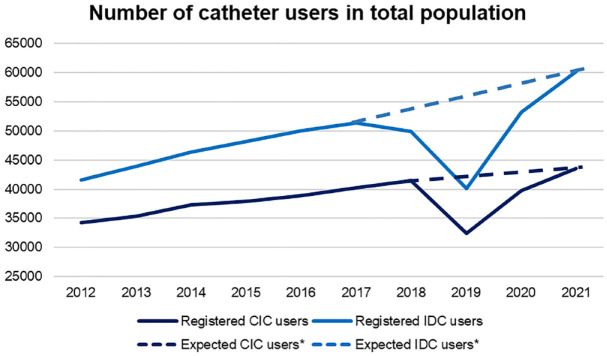
Number of catheter users corrected for the total population from 2012 to 2021. *In 2019 and 2020 the number of catheter users was underestimated due to missing data from health insurers. The expected number of users is higher without a decrease in IDC and CIC users over time. CIC, clean intermittent catheter; IDC, indwelling catheter

Between 2012 and 2021, the number of IDC users increased from 248 to 343 users per 100,000 people. In the same decade, the number of CIC users increased from 204 to 248 users per 100,000 people. The absolute number of IDC users increased by 44.6% from 41,619 to 60,172 users. The absolute number of CIC users increased by 27.3% from 34,204 to 43,528 users. Figure 1 shows the time trend of catheter users in the total population from 2012 to 2021. In 2019 and 2020, a decrease in the absolute number of IDC and CIC users was observed. This is due to missing records on catheter declarations from the Netherlands’ third-largest health insurer, the VGZ (Stichting Volksgezondheidszorg) Health Insurance group. In 2018, their percentage of records on catheter declarations was 4% of their total number of records. In 2019, this percentage decreased to almost 0%, before rising to 3% in 2020 and normalized to 5% in 2021. If we eliminate all the records of the VGZ group from the analyses, no decrease in the absolute number of IDC and CIC users is observed, but instead, a continuous increase is perceived in 2019 and 2020 (Figure 1).

**Figure 2.**
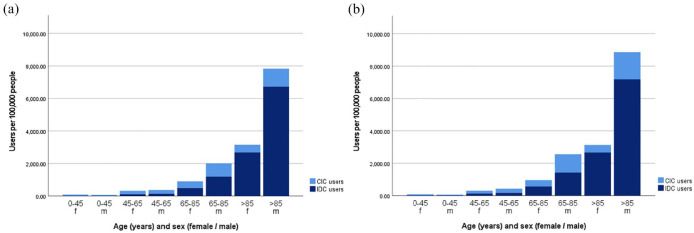
Age and sex distribution of CIC and IDC users per 100,000 people in 2012 (a) and 2021 (b). CIC, clean intermittent catheter; IDC, indwelling catheter.

Figure 2 presents the distribution of IDC and CIC users among different gender and age groups in 2012 and 2021. The number of male CIC users aged 65–85 and > 85 years increased the most by 39.2% (from 813 to 1131 users per 100,000 people) and 51.0% (from 1111 to 1677 users per 100,000 people), respectively. The number of male CIC users aged 45–65 years increased slightly by 7.5% from 231 to 248 users per 100,000 people. For all age categories of female CIC users, a small decrease in users was observed over the past 10 years. The number of female CIC users aged 0–45 and 45–65 years decreased by 10.4% (from 75 to 68 users per 100,000 people) and 16.7% (from 216 to 180 users per 100,000 people), respectively. The number of female CIC users aged 65–85 and > 85 years remained relatively stable, with 410–396, and 466–455 users per 100,000 people, respectively. For IDC users, the highest increase was among users aged 0–45 years, with an increase of 49.4% (from 11 to 17 users per 100,000 people) for male users and 56.8% (from 15 to 23 users per 100,000 people) for female users. However, the largest absolute increase was observed among men older than 85 years, from 6721 to 7185 users per 100,000 people. This is in contrast to female IDC users in the same age group, where no increase was observed (from 2685 to 2675 users per 100,000 people). What is evident in Figure 2 is the increased risk of catheterization as age increases, this applies to both IDC and CIC users.

### Catheter users: provinces

**Figure 3.**
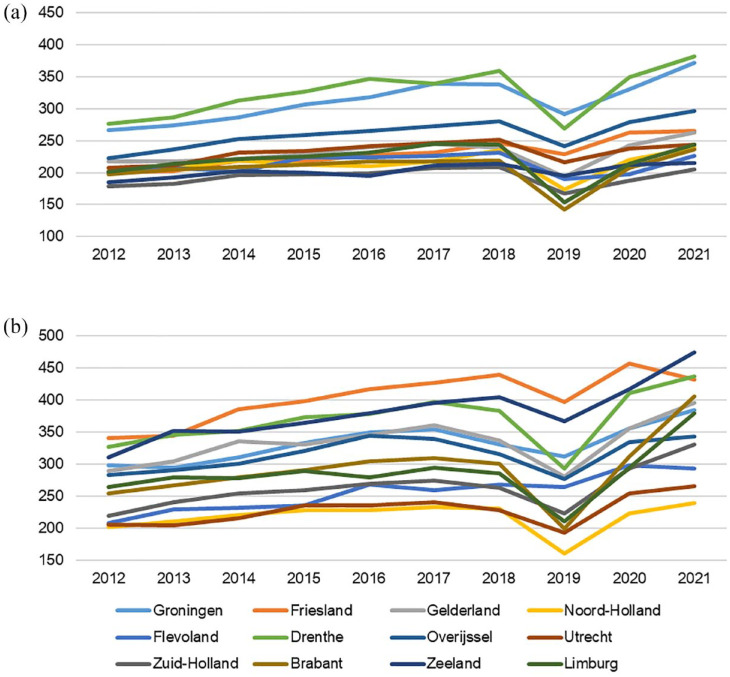
CIC users (a) and IDC users (b) per 100,000 people per province from 2012 to 2021. CIC, clean intermittent catheter; IDC, indwelling catheter.

Figure 3 shows the numbers of IDC and CIC users by province between 2012 and 2021. All provinces showed an increase in IDC and CIC users over the past 10 years. The largest increase in IDC users was seen in the provinces Brabant and Zeeland (Southern Netherlands), with an increase of 59% and 50% in the number of users, respectively. The largest increase in CIC users was seen in the provinces Groningen and Drenthe (Northern Netherlands) with an increase of 39% and 38% in the number of users, respectively. In addition, regional variation of IDC and CIC users has increased over time. In 2012, the number of IDC users per province ranged from 202 to 304 users per 100,000 people, compared with 239–474 users in 2021. Similarly, the number of CIC users per province ranged from 179 to 277 users per 100,000 people in 2012, compared with 205–382 users in 2021.

**Figure 4.**
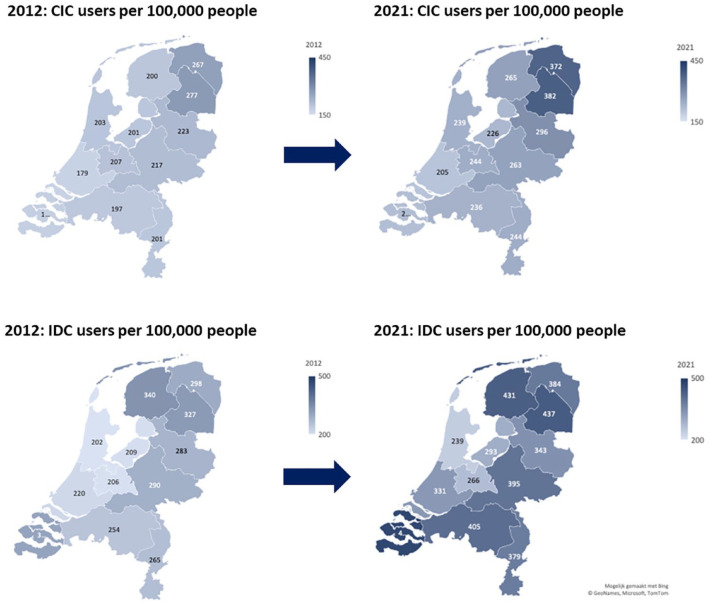
Differences in CIC and IDC users on the geographical map of the Netherlands between 2012 and 2021. CIC, clean intermittent catheter; IDC, indwelling catheter.

**Figure 5.**
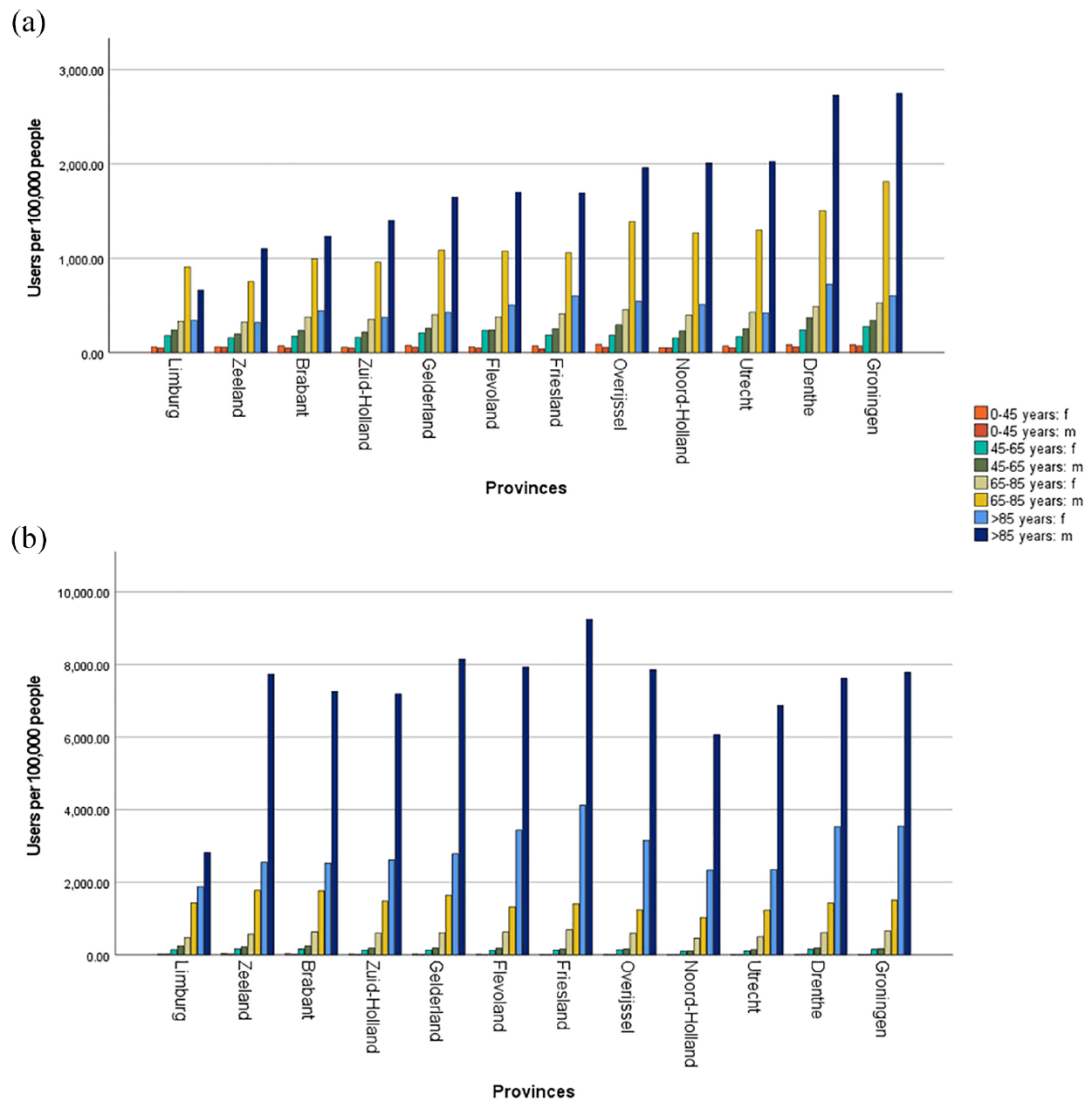
Age and sex distribution of CIC users (a) and IDC users (b) per province in 2021. CIC, clean intermittent catheter; IDC, indwelling catheter.

Figure 4 shows the differences in IDC and CIC users on the geographical map of the Netherlands between 2012 and 2021. The geographical map shows an increase in IDC and CIC users for all provinces. In 2021, a higher number of CIC users was seen in the northern part of the Netherlands compared to the other regions. A high number of IDC users was geographically more dispersed across the Netherlands (both north and south regions). The regional differences between the number of IDC users were determined by males and females older than 85 years [Figure 5(a)]. This contrasts with the regional differences among CIC users, which were mainly determined by males aged 65–85 years and 85 years and older [Figure 5(b)].

Multivariable NBR analyses showed significant differences between the 12 provinces for IDC and CIC users (Tables 1 and 2). The incidence of CIC users was higher in Drenthe and Groningen (Northern Netherlands) compared to Zuid-Holland when holding all other predictors constant (IRR = 1.61, 95% CI 1.17–2.21, *p* = 0.003 and IRR = 1.67, 95% CI 1.22–2.29, *p* = 0.002, respectively). The incidence of IDC users was higher in seven provinces dispersed throughout the Netherlands compared to the province Noord-Holland (Table 2). In addition, the rate of CIC and IDC users was higher among males compared with females (IRR = 1.80, 95% CI 1.57–2.06, *p* < 0.001 and IRR = 2.03, 95% CI 1.84–2.23, *p* < 0.001 respectively), and the older the age the higher the rate of IDC and CIC users. For example, people aged 85 and older were expected to use an IDC 28.43 times more often compared with people aged 45–65 years (*p* < 0.001).

**Table 1.** Results of NBR analysis for CIC users in 2021.

Parameter	IRR (95% CI)	*p* Value
Province (reference = Zuid-Holland)		
Zeeland	0.93 (0.68–1.27)	0.641
Limburg	0.93 (0.68–1.28)	0.677
Brabant	1.08 (0.79–1.48)	0.634
Noord-Holland	1.16 (0.84–1.59)	0.369
Friesland	1.18 (0.86–1.62)	0.302
Flevoland	1.18 (0.86–1.62)	0.301
Gelderland	1.21 (0.88–1.67)	0.230
Utrecht	1.22 (0.89–1.67)	0.218
Overijssel	1.36 (0.99–1.87)	0.054
Drenthe	1.61 (1.17–2.21)	0.003
Groningen	1.67 (1.22–2.29)	0.002
Sex (reference = female)		
Male	1.80 (1.57–2.06)	<0.001
Age (reference = 0–45 years)		
45–65 years	3.35 (2.79–4.03)	<0.001
65–85 years	10.37 (8.59–12.51)	<0.001
>85 years	13.97 (11.55–16.89)	<0.001

CIC, clean intermittent catheter; NBR, negative binomial regression; IRR, incidence rate ratio.

**Table 2.** Results of NBR analysis for IDC users in 2021.

Parameter	IRR (95% CI)	*p* Value
Province (reference = Noord-Holland)		
Utrecht	1.15 (0.91–1.45)	0.246
Limburg	1.17 (0.93–1.48)	0.176
Zuid-Holland	1.35 (1.07–1.70)	0.012
Overijssel	1.35 (1.07–1.71)	0.011
Gelderland	1.43 (1.13–1.80)	0.003
Flevoland	1.38 (1.10–1.74)	0.006
Groningen	1.47 (1.17–1.86)	0.001
Drenthe	1.50 (1.16–1.84)	0.001
Zeeland	1.50 (1.19–1.89)	<0.001
Brabant	1.50 (1.19–1.89)	<0.001
Friesland	1.50 (1.19–1.90)	<0.001
Sex (reference = female)		
Male	2.03 (1.84–2.23)	<0.001
Age (reference = 45–65 years)		
65–85 years	5.72 (5.09–6.43)	<0.001
>85 years	28.43 (25.28–31.98)	<0.001

IDC, indwelling catheter; NBR, negative binomial regression; IRR, incidence rate ratio.

## Discussion

Indwelling and intermittent catheters are widely used for bladder drainage in the treatment of urinary retention in neurogenic and non-neurogenic patients. This study explored the trends and regional differences in CIC and IDC users in the community setting in the Netherlands from 2012 to 2021. Our results showed that the number of CIC and IDC users has continued to increase in recent years. This is consistent with our two previous studies that evaluated the use of urinary catheters in the Netherlands with an overlapping cohort from 1997 to 2018.^11,12^ They found that the number of CIC users tripled and the number of IDC users doubled over two decades. Moreover, they found an increase in CIC and IDC use in the younger age categories, which may be a result of exponential population growth, among other factors. In comparison with our study, an increase in users was observed mainly among male IDC users older than 85 years and male CIC users older than 65 years. While, surprisingly, the number of female catheter users slightly decreased or remained stable over time. Differences between male and female catheter users were also found in a previous study of urinary catheter prevalence in England.^19^ They found a two times higher prevalence of male catheter users in the community setting. This contrasts with other commonly used medical devices by older people, such as hearing aids, where no gender differences were found.^20^ It is therefore reasonable to conclude that the increase in male catheter users appears to be due to urological factors.

First, compared to females, males have a longer urethra and are predisposed to prostate disease, which increases the likelihood of urinary retention and the use of catheters. Benign prostate hyperplasia (BPH) is an age-related disease that increases in prevalence as men get older. The number of men diagnosed with BPH has increased over the past decade, partly due to an ageing population with an increase in the number of older men and an increase in life expectancy.^21^ As people live longer, they are more likely to experience chronic health conditions and require long-term care. One common aspect of this care is the use of catheters, due to urinary retention or incontinence. Because the capacity of nursing homes and long-term care facilities is often limited, many seniors continue to live independently at home for longer. Some patients can perform CIC in old age, or they receive IDCs in the community setting due to high workloads and understaffing in the healthcare sector. In addition, the prevalence of urinary incontinence has increased in recent years partly due to an aging population.^22^ The Centers for Disease Control and Prevention’s National Center for Health Statistics in the United States reported an incontinence prevalence of 43.8% in the noninstitutionalized population older than 65 years.^23^ This increase and high prevalence may have contributed to the increased use of IDCs. According to the Dutch national GIP database, the use of incontinence materials (i.e. absorbent pads) ranked second among the most frequently used medical devices in 2022, with a total of 431,700 users, and third in terms of total medical device costs, with a total of 139 million euros.^24^ Unfortunately, incontinence materials are not always fully reimbursed in the Netherlands, and therefore there can be a financial incentive for patients to opt for reimbursed IDCs, despite that they have more side effects. As a result, the use of IDCs may continue to increase undesirably in the future.

In addition, the rising prevalence of prostate cancer may have contributed to the increasing use of catheters in older men. In the Netherlands, the prevalence of prostate cancer increased from 104,000 to 124,000 men between 2012 and 2022, of whom 78% were aged 60 years or older.^25^ Locally advanced prostate cancer can cause urethral obstruction, which may require (temporary) catheterization. In addition, prostate cancer treatment can lead to urinary complications that necessitate catheterization. For example, radiation therapy may cause urinary retention due to radiation-induced inflammation and scarring of the lower urinary tract, while radical prostatectomy may lead to urinary incontinence.^26,27^ These consequences of prostate cancer may thus have contributed to the increased use of catheters in older men.

The coronavirus disease 2019 (COVID-19) pandemic had massive consequences for society and healthcare during 2020 and 2021. One might hypothesize that COVID-19 might have brought substantial changes in urinary catheter utilization in the community setting, due to a notable shift towards home-based care, driven by both the necessity to minimize the risk of viral transmission in healthcare institutions and the need to preserve healthcare resources for the management of COVID-19 patients. However, we did not observe any changes in catheter utilization during this period. Van Deukeren *et al.* showed that the impact of the first COVID-19 wave on men with de novo prostate cancer in the Netherlands was limited and that the number of radical prostatectomies in 2020 was comparable to previous years.^28^ Only surgeries for benign diseases were postponed during this period, resulting in a decrease in the number of transurethral resections of the prostate by 1500 and 2500 procedures in 2020 and 2021, respectively.^29^ These small numbers did not affect the overall catheter use over time.

This is the first study describing regional differences of CIC and IDC users in the community setting in the Netherlands. Only one previous study reported on the prevalence of long-term IDC use in the community setting in different regions of England.^13^ They found no differences between the regions. In contrast, our findings showed that there were notable variations in the number of CIC and IDC users between different regions in the Netherlands. These results were rather unexpected, as we hypothesized that there were no regional differences in the type of catheter users across our relatively small country with a uniform urology and rehabilitation residency program and education program for continence nurses. The use of CIC was more prominent in the northern region of the Netherlands, while the use of IDC varied between multiple provinces across the country. These regional differences might be explained by multiple reasons.

First, it can be explained by differences in preferences of healthcare providers and their alignment with professional guidelines. According to the European Association of Urology (EAU) and American Urological Association (AUA) guidelines, CIC is considered the preferred method for bladder drainage in neurogenic and non-neurogenic patients.^7,8^ Since CIC was more prominent in the northern region of the Netherlands, it is possible that clinicians in the north are more likely to follow the guidelines than clinicians in the south. A previous study showed that only 53% of Dutch urologists used evidence-based guidelines for their practice in neurogenic lower urinary tract dysfunction and that some observations of their practice contradicted the recommendations of these guidelines.^30^ In addition, in the Netherlands, CIC is only prescribed by urologists and rehabilitation physicians while IDCs can also be prescribed by other healthcare providers (e.g. general practitioners, nurses, other specialists). This may also contribute to greater regional disparities among IDC users compared to CIC users.

Second, these regional differences can be explained by variations in patient populations, including contributing factors such as underlying health conditions, demographic characteristics, and cultural factors. Geographical disparities in the prevalence of neurogenic and non-neurogenic patients, as well as differences in the provision of specialized care, can lead to variations in catheter users across regions. Moreover, demographic factors, such as socioeconomic status, can vary significantly by region and thus may contribute to regional differences in catheter use. Especially, since incontinence materials are not always fully reimbursed and patients may prefer an IDC as a result. Cultural factors may also contribute to regional differences in catheter users. Cultural beliefs, practices, and attitudes towards healthcare interventions can influence the acceptance or refusal of CIC or IDC.^31^

Understanding regional differences in urinary catheter users is essential for healthcare providers and policymakers to develop targeted interventions. Identifying areas with lower rates of CIC use can help prioritize educational initiatives and promote evidence-based guidelines. Future interventions should focus on improving the standard of care for patients requiring urinary catheterization, such as standardizing a personalized decision-making process regarding assisted bladder drainage (e.g. a catheter decision aid).

The generalizability of these results is subject to certain limitations. In the Netherlands and most European Union countries, urinary catheters are reimbursed by healthcare insurance. Unfortunately, the availability of healthcare resources and reimbursement for urinary catheters is not always guaranteed, especially in low-income countries. These socioeconomic aspects may influence the choice of catheter type. For example, a study on the use of CIC among patients with spinal cord injury in Tanzania found that the majority of patients discontinued CIC after discharge from the hospital, due to unavailability of CIC equipment.^32^ Our results are, therefore, difficult to extrapolate to other countries with different availability of health care resources and reimbursement policies.

In addition, our study did not evaluate the use of urinary catheters in healthcare institutions, such as hospitals, rehabilitation facilities, or nursing homes. Consequently, the actual use of catheters is expected to be significantly higher, especially for IDCs.

Another limitation is that the GIP database only contains information on the number of catheter prescriptions. Data on the duration (e.g. chronic or temporary use) and indication of catheter use are lacking, whereas these data could help explain regional differences. One possible explanation could be that these regional differences come from differences in patient populations (e.g. neurogenic or non-neurogenic) and thus differences in the indications for initiating catheterization. Despite these limitations, this is the first study that evaluated regional differences in urinary catheter use in the community setting using a large population-based cohort and contributes to the limited knowledge of urinary catheter use in the Netherlands.

## Conclusion

Although the current study is limited to data from the community setting (non-hospitalized and non-institutionalized) in the Netherlands, the findings show that the number of indwelling and intermittent urinary catheter users has continued to increase over the past decade. Interestingly, this increase is mainly observed in older men and not in women. This may be related to the fact that men have a longer urethra and are prone to age-related prostate diseases (i.e. BPH, prostate cancer), making them more susceptible to urinary retention. In addition, there are notable differences in the number of CIC and IDC users among different regions in the Netherlands. The use of CIC is more prominent in the northern region of the Netherlands, while the use of IDC varies between multiple provinces across the country. Differences in practice regarding urinary catheterization might be explained by differences between patient populations, or differences between the preferences of healthcare providers (urologists/rehabilitation physicians/general practitioners) and their alignment with professional guidelines. The findings of this study emphasize the need to focus on future interventions to improve the standard of care for patients requiring urinary catheterization, such as the standardization of a personalized decision-making process regarding assisted bladder drainage.

## Declarations

**Ethics approval and consent to participate:** Formal ethical approval was not required, as all the study data were obtained from a nationwide anonymized database, which is publicly available. We requested a linking of the dataset and this link was made by the governmental institution and the total (anonymized) results were shared with us.

**Consent for publication:** Not applicable.

**Availability of data and materials:** The datasets generated and analyzed during the current study are available from the corresponding author on reasonable request.
